# Redefining koinophilia: Solution to social isolation and polarization

**DOI:** 10.1073/pnas.2537378123

**Published:** 2026-02-18

**Authors:** Chika Edward Uzoigwe

**Affiliations:** ^a^Department of Medicine and Surgery, Harcourt House, Sheffield S10 1DG, United Kingdom

Thurner et al. present an intriguing, ethereal, and empirical study on the link between connectedness and social polarization (1). However the authors’ work is abstract and potentially underplays the tangible media and social baupläner by which connectedness occurs, but yet polarization transpires. This introduces an important but overlooked confounder. It is well established that the increasing trend toward urbanization leads to increasing connectivity but greater segregation and polarization (2–4). In a work, equally as seminal as that of the Thurner group, Nilforoshan et al. found a direct correlation between municipal population size and social segregation. Rather than the dissonance paradigm advanced by Thurner’s team, the authors found that larger populations allowed people to seek, find, and socially sojourn with those with a socioeconomic commonality, whereas such options are not possible, to the same degree, in smaller conurbations. Indeed, the larger the population, the more narrow the social tranches that crystalized. Rather than homophily per se, this is more a social koinophilia, where there is a predilection to seek those who share a commonality or satisfy our perception of median social traits (5). This actually promotes and stabilizes preexistent distinct polarities (6). Critically, persons, when they socially engage, are not conceptually naive, but have established beliefs and values. This is more than an academic difference; it is germane to the solution of social polarization. Thurner and collaborators posit increased tolerance to opposing views as the remedy. Tolerance is laudable and worthy of pursuit in its own right. However, given the foregoing this “teletolerance” or tolerance at a distance may not address segregation and polarization. Nilforoshan et al. found that social isolation was ameliorated when common amenities such as shopping centers, plazas, cinematic, sporting, and entertainment complexes were placed at the intersections of segregated communities (2). In a similar vein, Renninger et al. also very recently identified the cardinal medium of social isolation and polarization was the concentration of amenities in a city bauplan that potentiated segregation and disconnectedness; such amenities are located separately within socially immiscible quarters, rather than in the interfacial zones between segregated districts. Thus, the solution essentially involves redefining the *koinos* or commonality along which we aggregate. Hence, humans often segregate on the basis of different commonalities but can equally associate on the basis of common or universal commonalities, especially if strategically interposed between nodes of segregation.

In an iconic song, by the American band *Deep Blue Something*, a couple experience social dissonance. To resolve the issue, they realize that a commonality they share is a fondness for the iconic film “*Breakfast at Tiffany’s*” (7). *Escape (The Piña Colada) Song* by Rupert Holmes expresses a similar polemic-solution dyadic heuristic (8). Hence, the solution is to find a common platform. If commonality is established, any observed divergence or subsequent polarization of opinion is neither necessarily acrimonious nor fatal to a henotic existence.

## References

[1] S. Thurner, M. Hofer, J. Korbel, Why more social interactions lead to more polarization in societies. Proc. Natl. Acad. Sci. U.S.A. **122**, e2517530122 (2025). 41171853 10.1073/pnas.2517530122 PMC12595431

[2] H. Nilforoshan , Human mobility networks reveal increased segregation in large cities. Nature **624**, 586–592 (2023), 10.1038/s41586-023-06757-3. 38030732 PMC10733138

[3] O. Christ , Contextual effect of positive intergroup contact on outgroup prejudice. Proc. Natl. Acad. Sci. U.S.A. **111**, 3996–4000 (2014). 24591627 10.1073/pnas.1320901111 PMC3964129

[4] A. Renninger, N. O’Clery, E. Arcaute, US cities are defined by rings and pockets with limited socioeconomic mixing. Nat. Cities **2**, 1172–1182 (2025).

[5] J. H. Koeslag, P. D. Koeslag, Koinophilia. J. Theor. Biol. **167**, 55–65 (1994). 8176954 10.1006/jtbi.1994.1049

[6] J. H. Koeslag, Koinophilia groups sexual creatures into species, promotes stasis, and stabilizes social behaviour. J. Theor. Biol. **144**, 15–35 (1990). 2200930 10.1016/s0022-5193(05)80297-8

[7] J. B. Young, Truman Capote as a poet. Methodist Debakey Cardiovasc. J. **19**, 104–106 (2023). 37547890 10.14797/mdcvj.1270 PMC10402784

[8] R. Achs, *Hypocritical blame is unfitting* (Oxford Studies in Metaethics, 2024), **vol. 19**, p. 326.

